# Prenatal screening for trisomy 21: a comparative performance and cost analysis of different screening strategies

**DOI:** 10.1186/s12884-020-03394-w

**Published:** 2020-11-23

**Authors:** Tianhua Huang, Clare Gibbons, Shamim Rashid, Megan K. Priston, H. Melanie Bedford, Ellen Mak-Tam, Wendy S. Meschino

**Affiliations:** 1 Genetics Program, North York General Hospital, 4001 Leslie Street, Toronto, Ontario M2K 1E1 Canada; 2 Prenatal Screening Ontario, Better Outcomes Registry & Network (BORN) Ontario, Ottawa, Ontario Canada; 3 Department of Obstetrics and Gynecology, University of Toronto, Toronto, Ontario Canada; 4 Department of Molecular Genetics, University of Toronto, Toronto, Ontario Canada; 5 Department of Paediatrics, University of Toronto, Toronto, Ontario Canada

**Keywords:** Trisomy 21, Multiple marker prenatal screening, Non-invasive prenatal screening, Cell-free fetal DNA screening, Cost and performance

## Abstract

**Background:**

Prenatal screening for chromosome aneuploidies have constantly been evolving, especially with the introduction of cell-free fetal DNA (cfDNA) screening in the most recent years. This study compares the performance, costs and timing of test results of three cfDNA screening implementation strategies: contingent, reflex and primary.

**Methods:**

We modelled enhanced first trimester screening (eFTS) as the first-tier test in contingent or reflex strategies. cfDNA test was performed contingent on or reflex from eFTS results. A comparison was made between cfDNA screening using sequencing technology and Rolling Circle Amplification (RCA)/imaging solution. All model assumptions were based on results from previous publications or information from the Ontario prenatal screening population.

**Results:**

At an eFTS risk cut-off of ≥1/1000, contingent and reflex cfDNA screening have the same detection rate (DR) (94%) for trisomy 21. Reflex cfDNA screening using RCA/Imaging solution provided the lowest false positive rate and cost. The number of women requiring genetic counselling and diagnostic testing was significantly reduced and women received their cfDNA screening result 9 days sooner compared with the contingent model. While primary cfDNA screening improved the trisomy 21 DR by 3–5%, it was more costly and more women required diagnostic testing.

**Conclusion:**

Reflex cfDNA screening is the most cost-effective prenatal screening strategy. It can improve the efficiency of prenatal aneuploidy screening by reducing the number of patient visits and providing more timely results.

## Background

Prenatal screening for common chromosome aneuploidies has been a part of routine prenatal care in developed countries since the early 1990’s. Conventional prenatal screening tests, also known as multiple marker screening (MMS), measure maternal serum biochemical markers and, if performed in the first trimester, the nuchal translucency (NT) ultrasound marker. MMS is used to estimate the risk for trisomy 21, 18 and 13 in the first or second trimesters of pregnancy [1, 2].

In recent years, cell-free fetal DNA (cfDNA) screening has been introduced into clinical use. cfDNA screening assesses the risk for aneuploidy in the fetus by measuring cell-free fetal DNA in maternal blood [3]. The most commonly used cfDNA tests use sequencing-based technologies such as whole genome or targeted sequencing. Whole genome sequencing involves whole genome assay with next generation sequencing [3]. Targeted tests require only sequencing of specific regions of clinical relevance, and therefore have a lower cost, reduced assay variability and shorter analysis time. However, it uses complex genetic analysis platforms requiring a relatively advanced laboratory setup [4, 5]. Sequencing-based cfDNA analysis for trisomy 21 aims to determine the proportion of sequenced plasma DNA molecules originating from chromosome 21 relative to other chromosomes, as this proportion is expected to be elevated in maternal plasma in pregnancies with a trisomy 21 fetus [3]. This cfDNA method has a detection rate (DR) of about 99% and a false positive rate (FPR) of about 0.1% for trisomy 21 compared to a DR of 85–87% and FPR of 3% provided by the first trimester combined screening (FTS) [6–8]. It also detects about 91–96% of trisomy 18 and 13 cases for a FPR of about 0.12% [6]. cfDNA screening is however significantly more costly than MMS. Additionally, with sequencing- based cfDNA screening, about 2–3% of the women will receive a no-call result with low fraction of fetal DNA being the most common reason [6, 9]. cfDNA screening technologies are quickly evolving. For example, a testing platform using automated high precision Rolling Circle Amplification (RCA)/Imaging solution has been reported recently. This test uses novel molecular probe technology that specifically labels target chromosomes and digital molecular quantification without DNA amplification, microarrays or sequencing [5, 10]. It provides a similar DR to the sequencing-based cfDNA screening but with a lower no-call rate since the technology is less influenced by the fetal fraction [10]. It is also fully automated, less expensive than sequencing based tests and can be implemented in a non-molecular laboratory setting [5].

cfDNA screening has been used as a secondary test contingent on the MMS result or as a primary test in some screening programs [11, 12]. Reflex cfDNA screening was reported recently as a new approach for combining MMS and cfDNA screening [13, 14]. With reflex cfDNA screening, in contrast to contingent cfDNA screening, only a single final screening result is issued, either the first-tier MMS result if screen negative, or a cfDNA screening result if screen positive for MMS. With this strategy, 11% of samples are reflexed to cfDNA screening, the overall DR for trisomy 21 is 95% and FPR is as low as 0.02% [13]. While there are multiple publications on the cost and performance of primary and contingent cfDNA screening, only one study has focused on the cost and effectiveness of a reflex cfDNA screening strategy [15–17].

Since 2014, cfDNA screening has been publicly funded in Ontario for a subset of women with an increased risk in pregnancy for the common aneuploidies. The test indications include positive MMS, age ≥ 40 years at expected date of delivery, NT ≥ 3.5 mm and/or other aneuploidy risk factors in pregnancy [18]. To improve the accuracy of first-tier MMS, enhanced FTS (eFTS) was introduced in 2016 using NT measurement, first trimester pregnancy-associated plasma protein A (PAPP-A), free-β human chorionic gonadotrophin (hCG), placental growth factor (PlGF), α-fetoprotein (AFP) and maternal age [8]. Using both published data and data observed from Ontario’s population, the current study compares the performance and costs of various aneuploidy screening strategies incorporating cfDNA testing.

## Methods

In this study we modelled contingent, reflex and primary cfDNA screening strategies and compared their performance, cost and test turnaround time for trisomy 21 screening. For contingent and reflex cfDNA screening scenarios, enhanced FTS was used as the first-tier test. Currently in Ontario, an eFTS positive result for trisomy 21 is defined as a risk ≥1 in 350 at term. For the purpose of this study, eFTS positive was defined as a risk of ≥1 in 1000 at term in order to maximize case detection. In a contingent cfDNA screening model, the eFTS result is reported after the test has been completed and a decision made about follow-up testing after discussion with the woman’s health care provider. In a reflex cfDNA screening model, two blood samples are collected from every woman at their initial blood draw for aneuploidy screening. One is used for eFTS and the other reserved for cfDNA screening if the eFTS is screen positive. Only a single test result is issued; either a negative eFTS result or a cfDNA screening result for eFTS positive women. In a primary cfDNA screening model, all women have cfDNA screening as a first line test and eFTS test is not performed. For the reflex and primary cfDNA screening models, we compared cfDNA screening using the RCA/Imaging solution (as an example of an emerging cfDNA screening technology) to the currently available sequencing-based cfDNA screening technologies. cfDNA screening with RCA/Imaging solution was chosen because of its reported short turnaround time, low no-call result rate and suitability for use in any biochemical laboratory [5]. For reflex cfDNA screening using RCA/Imaging solution, we also modelled performance and cost using an eFTS two-risk cut-off, with women with an eFTS risk of ≥1 in 50 having a prenatal diagnostic test and those with an intermediate risk of ≥1 in 1000 but < 1 in 50 having cfDNA screening. The outputs for the different screening strategies were calculated using Microsoft Excel 2016 (Microsoft Corp.).

**Fig. 1:**
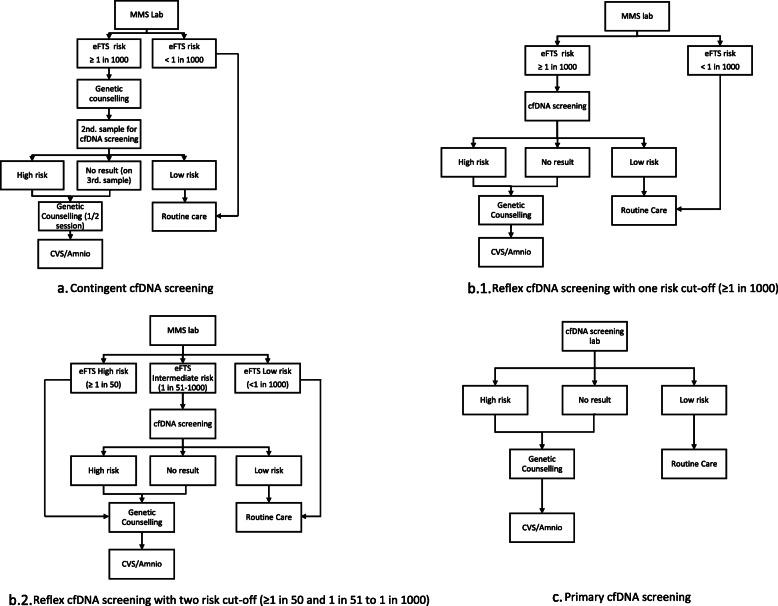
Prenatal screening pathway using different screening strategries. eFTS: Enhanced first trimester screening; cfDNA screening: cell free fetal DNA screening; Amnio: Amniocentesis, CVS: Chorionic villus sampling

Figure 1 shows the screening pathways for the contingent, reflex and primary cfDNA screening models. Summarized in Table 1 are the assumptions used in the modelling calculations, including about the screening population, the performance, turnaround time, and unit cost of eFTS and cfDNA screening, genetic counselling (GC) time and prenatal diagnostic testing. We assumed 100,000 women will be screened each year, a number similar to the annual screen volume in our previous publication [17]. The following measures were taken from previous publications: age-specific prevalence of trisomy 21, overall prevalence of trisomy 21 in the study population, spontaneous fetal loss rate of trisomy 21-affected pregnancies in the first trimester, procedure-related fetal loss rate of unaffected pregnancies, DR and FPR of eFTS (at a risk cut-off of 1 in 1000) and cfDNA screening, no-call result rates of cfDNA screening, and the unit cost of eFTS and amniocentesis/CVS [5, 6, 8, 9, 15, 17, 19–21]. The screening performance of eFTS and cfDNA used was based on singleton pregnancies including women who conceived through in vitro fertilization. DR and FPR of eFTS using a two-risk cut-off model (high risk of ≥1 in 50 and intermediate risk of≥1 in 1000 but < 1 in 50) were based on unpublished data obtained through personal communication with Professor Howard Cuckle (on April 15, 2018). The median gestational age at eFTS, and the turnaround times of eFTS and GC were estimated based on women screened at one large Ontario screening centre which performs about 45% of the MMS tests in Ontario. The turnaround time of 10 business days for sequencing-based cfDNA screening was based on local experience and on the websites of the two providers offering funded cfDNA screening in Ontario [22, 23]. We chose 4 business days as the turnaround time of cfDNA screening using RCA/Imaging solution for the modelling calculation. This was estimated based on a turnaround time of 4–7 days (including weekend) from personal communication with Professor Geralyn Messerlian (on September 23, 2020) and a white paper from the company developing the test (which reported a shorter turnaround time of 2 days). The 4–7 days turnaround time includes 3 full days for sample analysis and 1 day for data transfer to reporting file. The cost of blood collection tubes for cfDNA screening was based on local estimation. The unit cost of genetic counselling was estimated from Physician Services under the Health Insurance Act, Schedule of Benefits (Page A67), the Ministry of Health and Long-term Care, Ontario (December 2015). We assumed a unit cost of $400 CAD for both sequencing-based cfDNA screening and cfDNA screening using RCA/Imaging solution to streamline the comparison. This cost was estimated based on the cost of sequencing-based cfDNA screening for the common aneuploidies available to self-pay patients in Ontario and after taking into account a possible future price drop [22, 23].

**Table 1:** Assumptions on prenatal screening for trisomy 21

Parameter	Assumptions	
***Background***		
Number of pregnancies screened [17]	100,000	
Prevalence of T21 [19]	0.26%	
Spontaneous fetal loss rate of T21 [20]	43.0%	
Fetal loss rate after amnio/CVS [21]	0.30%	
Median GA at eFTS (days)	88	
***Test performance***		
eFTS risk cut-off	1 in 1000 (a)	1 in 50 and 1 in 51–1000 (b)
eFTS DR [8]	95% (a)	67 and 28% (b)*
eFTS FPR [8]	11% (a)	1 and 10% (b)*
cfDNA screening DR [5, 6, 10]	99%	
cfDNA screening FPR [5, 6, 10]	0.1%	
cfDNA screening no-call result of 1st/2nd sample [5, 9]	3.3% /1.2% (c)	0.5%/NA (d)^#^
***Turnaround time (days)***		
Sample received at MMS lab to eFTS result issued	3	
Sample received at cfDNA screening lab to cfDNA screening result issued [22–24]	10 (c)	4 (d) ^+^
Positive eFTS, high risk or no-call cfDNA screening to GC	3	
***Cost (CAD)***		
Unit cost of blood sample collection tube for cfDNA screening	$10	
Unit cost of eFTS [17]	$120	
Unit cost of cfDNA screening [ 22, 23]	$400 ^@^	
Unit cost of GC	$250 ^^^	
Unit cost of CVS/amnio [17]	$1786.9	

*T21* Trisomy 21, *CVS* Chorionic villus sampling, *Amnio* Amniocentesis, *GA* gestation age, *eFTS* Enhanced first trimester screening, *DR* Detection rate, *FPR* False positive rate, *cfDNA screening* cell free fetal DNA screening, *RCA/Imaging solution* automated high precision rolling circle amplification/Imaging solution, *GC* Genetic counselling, *PND* Prenatal diagnosis testing

(a) One eFTS risk cut-off; (b) Two eFTS risk cut-offs; (c) Sequencing based cfDNA screening, (d) cfDNA screening using RCA/Imaging solution

* Personal communication with Professor Howard Cuckle based on unpublished data from a study on eFTS performance for trisomy 21; ^#^ Round up from 0.28%; ^+^ Personal communication with Professor Geralyn Messerlian; ^@^ The cost of a basic cfDNA test to self-paid patients after considering possible future price drop for both sequencing and RCA/Imaging based tests; ^ Estimated from Physician Services Under the Health Insurance Act, Schedule of Benefits (Page A67), Ministry of Health and Long-term Care, December 22, 2015

For the contingent cfDNA screening strategy, women receiving a positive eFTS result would be offered genetic counselling as is the current practice in Ontario. A second blood sample would be required for follow-up cfDNA screening. If the cfDNA screening provides no result, a blood test for A repeat cfDNA screening would be offered (a third blood sampling). A half-session of genetic counselling (counselling and Q & A by phone) and a diagnostic test would be offered following either a high risk cfDNA screening result or no-call result on repeat cfDNA screening (Fig. 1a). For reflex cfDNA screening using a single eFTS risk cut-off (1 in 1000), no repeat cfDNA screening would be performed for a no-call result. A genetic counselling session and a prenatal diagnostic test would be offered to women with either a high risk or no-call cfDNA screening result (Fig. 1b.1). When using an eFTS two-risk cut-off, a genetic counselling session and prenatal diagnostic testing would be offered to women with either a high eFTS risk (≥ 1 in 50) or those with a high risk or no-call on cfDNA screening following an intermediate eFTS risk (≥1 in 1000 but < 1 in 50) (Fig. 1b.2). For primary cfDNA screening, repeat cfDNA screening would be offered to women who have a no-call result from sequencing-based cfDNA screening, but not from the cfDNA screening with RCA/Imaging solution technology given the test’s low no-call rate [5]. A genetic counselling session and prenatal diagnostic testing would be offered to women receiving a high risk or no-call cfDNA screening result (Fig. 1c). In screening practice, the uptake of genetic counselling, contingent cfDNA screening, and diagnostic testing is variable across populations. In this study, we attempted to maximize performance and streamline comparison between the different screening models by assuming 100% uptake of genetic counselling services and follow-up testing after receiving either a positive eFTS or a high risk or no-call cfDNA screening result.

The performance measures of each screening strategy include the number of trisomy 21 affected pregnancies diagnosed prenatally, number of trisomy 21 affected pregnancies missed by the screening strategy, number of live births with trisomy 21, number of procedure- related fetal losses, number of genetic counselling sessions, number of prenatal diagnostic tests, and DR and FPR. The cost measures include the total program cost, and cost per woman screened, per case of trisomy 21 diagnosed prenatally and per additional case of trisomy 21 diagnosed prenatally, when compared with the current contingent screening strategy. The turnaround time and timeliness of the screening result was measured by number of days from initial blood sample draw to cfDNA screening result reported and number of visits needed to receive a cfDNA screening result. The cost and performance for trisomy 18 and 13 screening were not modelled separately in this study as most of the pregnancies affected with trisomy 18 or 13 would be screen positive for trisomy 21 [25]. The eFTS positive rate for trisomy 18 or 13 alone is very low and has minimal impact on the number of women requiring secondary cfDNA screening or diagnostic testing.

The Research Ethics Board of North York General Hospital ruled that no formal ethics approval was required in this particular study.

## Results

Assuming 100,000 women are screened in Ontario annually, Table 2 compares screening performance for trisomy 21 with contingent, reflex and primary cfDNA screening. When using sequencing-based cfDNA screening, the DR of contingent and reflex cfDNA screening are the same at 94.1%. Since no repeat cfDNA screening is offered with reflex cfDNA screening, 230 more women would need a diagnostic test with this strategy (if all women receiving a no-call cfDNA screening result undergo a diagnostic test). However, the number of genetic counselling sessions is substantially reduced from 11, 443 with contingent cfDNA screening to 618 with reflex cfDNA screening. Using sequencing-based cfDNA screening as the primary screening test, the DR is higher at 99%, but the real ‘FPR’ is much higher at 1.3% if all women with a no-call result have a diagnostic test. If women with a no-call result are excluded from analysis, 254 of 260 cases of trisomy 21 can be detected providing a DR of 97.7% for a FPR of 0.1%. To achieve a DR of 99%, 1554 women would need a diagnostic test with primary sequencing-based cfDNA screening compared to 388 with contingent cfDNA screening for a DR of 94.1%. Primary sequencing-based cfDNA screening is associated with 4 more cases of procedure-related fetal loss of an unaffected fetus than the contingent model.

**Table 2:** Screening performance for trisomy 21 using contingent, reflex, and primary cfDNA screening strategies (assuming a screening population of 100,000 women, and eFTS used as the primary screening test for contingent and reflex cfDNA screening)

Strategy	Contingent cfDNA screening	Reflex cfDNA screening			Primary cfDNA screening	
Risk cut-off(s) of eFTS	1 in 1000	1 in 1000	1 in 1000	1 in 50 and 1 in 1000		
cfDNA screening technology	Sequencing	Sequencing	RCA/Imaging solution	RCA/Imaging solution	Sequencing	RCA/Imaging solution
# eFTS positive	11,250	11,250	11,250	11,250 (a)	–	–
# cfDNA screening	11,250	11,250	11,250	9850	100,000	100,000
# cfDNA screening high risk	253 (b)	247 (c)	254 (c)	82 (c)	354 (b)	356 (c)
# cfDNA screening no-call result	135 (d)	371 (e)	56 (e)	49 (e)	1200 (d)	500 (e)
# patient receiving GC	11,250 (f)	618 (g)	311 (g)	1532 (h)	1554 (g)	856 (g)
# GC session	11,443 (i)	618 (g)	311 (g)	1532 (h)	1554 (g)	856 (g)
# PND (cfDNA screening high risk)	253	247	254	1482 (j)	354	356
# PND (cfDNA screening high risk/no-call)	388	618	311	1532	1554	856
# Expected T21	260	260	260	260	260	260
# T21 diagnosed through PND (cfDNA screening high risk)	242	236	243	246 (k)	254	256
# T21 diagnosed through PND (cfDNA screening high risk/no-call)	245	245	245	246	257	257
# T21 eFTS negative	13	13	13	13	–	–
# T21 cfDNA screening low risk	2	2	2	1	3	3
# T21 cfDNA screening no-call	3	8	1	0	3	1
# T21 missed by eFTS or cfDNA screening	15	15	15	14	3	3
# T21 livebirth	9	9	9	8	2	2
# Procedure related fetal loss	0	1	0	4	4	2
DR (cfDNA screening high risk)	92.2%	91.0%	93.6%	94.6%	97.7%	98.5%
FPR (cfDNA screening high risk)	0.01%	0.01%	0.01%	1.24%	0.10%	0.10%
Overall DR (all cfDNA screening no-call have a PND)	94.1%	94.1%	94.1%	94.7%	99.0%	99.0%
Overall FPR (all cfDNA screening no-call have a PND)	0.14%	0.37%	0.07%	1.29%	1.30%	0.60%

Numbers of pregnancies were rounded to the nearest whole number

*T21* Trisomy 21, *cfDNA screening* cell free fetal DNA screening, *eFTS* Enhanced first trimester screening, *RCA/Imaging solution* automated high precision rolling circle amplification/Imaging solution, *GC* Genetic counselling, *PND* Prenatal diagnosis testing (Chorionic villus sampling and amniocentesis), *DR* Detection rate, *FPR* False positive rate

a. Women with eFTS risk≥1/50 (1400) or risk between 1/51–1/1000 (9850); b. cfDNA screening high risk from the first and second tests; c. cfDNA screening high risk from the first test (assuming no repeat test is offered if the first test has a no-call result); d. cfDNA screening no-call result after the second test (assuming a repeat test is offered if the first test has no-call result); e: cfDNA screening no-call result after the first test; f: Women screen positive for eFTS; g: Women with cfDNA screening high risk or no-call results; h: Women with eFTS risk≥1 in 50 (1400) and women with high risk (82) or no-call cfDNA screening results (49); i: Women with positive eFTS (11,250) have a full session of GC, women with a high risk cfDNA screening (253) and no-call cfDNA screening (135) also have a follow-up 1/2 session of GC; j: Women with eFTS risk≥1 in 50 (1400) and high risk cfDNA screening (82); k: T21 diagnosed among women with eFTS risk≥1 in 50 or high risk cfDNA screening

Using cfDNA screening with RCA/Imaging solution in a reflex strategy, the DR remains the same at 94.1%. The FPR is halved to 0.07% compared to contingent cfDNA screening using sequencing based cfDNA screening because of the reported lower no-call result rate of RCA/Imaging solution technology. The number of women requiring genetic counselling is reduced by over 95% (from 11,250 to 311) and the number requiring diagnostic testing is reduced by 20% (from 388 to 311). An eFTS two-risk cut-off strategy which offers diagnostic testing to women with high risk eFTS and cfDNA screening to those with an intermediate risk increases the DR for trisomy 21 only slightly to 94.7% (less than 1%). However, the overall FPR is 18-fold higher at 1.29%. The number of women requiring genetic counselling and diagnostic testing is 4-times higher compared to reflex cfDNA screening using a single risk cut-off of 1 in 1000. When comparing single risk cut-off reflex cfDNA screening using sequencing-based technology to the RCA/Imaging solution, the latter requires fewer genetic counselling sessions and diagnostic tests due to its lower no-call result rate. Primary cfDNA screening using the two technologies have the same DR (99%). However, the FPR and the number of women requiring genetic counselling and diagnostic testing are reduced by about 45% with cfDNA screening using RCA/Imaging solution.

Table 3 shows the costs associated with each screening strategy. Of the strategies using sequencing-based cfDNA screening, reflex cfDNA screening testing is the least costly for total program cost and cost per case of trisomy 21 diagnosed prenatally. Of all the reflex cfDNA screening scenarios, cfDNA screening with RCA/Imaging solution reflexed from eFTS with a single risk cut-off of ≥1 in 1000 has the lowest cost. Of the reflex cfDNA screening strategies, using of a two-risk eFTS cut-off has the highest cost. Primary cfDNA screening would cost double the amount of contingent or reflex cfDNA screening, even taking into account the lower total program cost of primary cfDNA screening with RCA/Imaging solution compared to the sequencing-based cfDNA technologies. With primary cfDNA screening, the cost for each additional case of trisomy 21 diagnosed when compared with contingent cfDNA screening is between 1.69 and 1.80 million dollars Canadian.

**Table 3:** Costs, number of patient visits and test turnaround time of different screening strategies

Strategy	Contingent cfDNA screening	Reflex cfDNA screening			Primary cfDNA screening	
Risk cut-off(s) of eFTS	1 in 1000	1 in 1000	1 in 1000	1 in 50 and 1 in 1000		
cfDNA screening technology	Sequencing	Sequencing	RCA/Imaging solution	RCA/Imaging solution	Sequencing	RCA/Imaging solution
**Costs (CAD)**						
Total program	$20,053,297	$18,759,518	$18,132,468	$20,059,786	$43,165,976	$41,743,817
Per woman screening	$201	$188	$181	$201	$432	$417
Per case of T21 diagnosed prenatally	$82,005	$76,529	$74,112	$81,169	$161,305	$159,510
Per case of T21 diagnosed prenatally (all cfDNA screening no-call have a PND)	$81,998	$76,691	$74,149	$81,455	$167,680	$162,167
Per additional case of T21 diagnosed compared to the contingent cfDNA screening	–	–	–	$3799	$1,795,639	$1,687,537
**Number of patient visit and turnaround time**						
Number of visit (1st cfDNA screening has result)	3	1	1	1	1	1
Number of visit (1st cfDNA screening no-call result)	4	1	1	1	2	1
Days from initial sample received to cfDNA screening result (1st cfDNA screening has result)	16	13	7	7	10	4
Days from initial sample received to cfDNA screening result (1st cfDNA screening no-call result)	26	13	7	7	20	4

*cfDNA screening* cell free fetal DNA screening, *eFTS* Enhanced first trimester screening, *RCA/Imaging solution* automated high precision rolling circle amplification /Imaging solution, *T21* Trisomy 21, *PND* Prenatal diagnosis testing

Table 3 also illustrates the number of patient visits and duration in days from initial blood sample received at laboratory to cfDNA screening result reported. For contingent cfDNA screening, 3–4 visits are needed for a patient to receive a final cfDNA screening result depending on whether the initial cfDNA screening result was a no-call or not. This includes the visits for eFTS sample draw, followed by separate visits for genetic counselling and cfDNA screening sample draw in eFTS positive women. With primary sequencing based cfDNA screening, 2 patient visits are needed for a woman whose first sample is a no-call result. All other strategies, including reflex cfDNA screening, requires only 1 patient visit. Primary cfDNA screening with the RCA/Imaging solution technology has the shortest turnaround time (4 days), followed by reflex cfDNA screening using this technology (7 days) and reflex sequencing-based cfDNA screening (13 days). This is compared to the contingent strategy using sequencing-based cfDNA screening which has the longest turnaround time (16–26 days). Assuming a median gestational age at initial blood sample draw of 88 days as observed in our screening population, the earliest gestational age receiving a cfDNA screening result is 92 days with primary cfDNA screening using RCA/Imaging solution, followed by reflex cfDNA screening using this technology (95 days). The latest gestation is contingent cfDNA screening using sequencing based cfDNA screening (104–114 days).

## Discussion

Our study models the performance, cost and timeliness of various screening strategies which incorporate cfDNA technology in a large prenatal population. While model assumptions such as turnaround time and unit costs of tests and services were based on Ontario numbers, the methodology of assessment can be used by other screening programs. Our results show that when cfDNA screening is used in high risk pregnancies identified by eFTS (i.e. risk≥1 in 1000), reflex cfDNA screening is the most cost-effective approach, especially if cfDNA screening with RCA/Imaging solution technology is used. For the same DR of 94% as with the current contingent cfDNA screening approach, reflex cfDNA screening with RCA/Imaging solution technology is associated with the lowest FPR and cost, and significantly reduces the number of women requiring genetic counselling and diagnostic testing. Moreover, these patients will receive cfDNA screening results 9 days sooner than with the current contingent sequencing-based cfDNA screening model. There is limited value in regards to trisomy 21 detection rate if women with a high risk eFTS result (≥ 1 in 50) are offered diagnostic testing over cfDNA screening as a follow-up test. This approach, however, may enable the diagnosis of other anomalies as suggested by previous publications [26, 27]. In this study, we used cfDNA screening with RCA/Imaging solution technology to model reflex cfDNA screening with eFTS two-risk cut-off as this technology is emerging as one with a shorter turnaround time, lower no-call rate, and therefore more suitable for reflex strategy. If sequencing-based cfDNA test is used, the DR will be the same. However, more women will need a diagnostic test because of its higher no-call rate. While primary cfDNA screening can improve DR for trisomy 21 by about 5%, the number of women requiring diagnostic testing is substantially increased, resulting in more procedure-related losses of unaffected fetuses. Introducing primary cfDNA screening would double the screening program cost compared to either the contingent or reflex cfDNA screening strategies.

Numerous studies have assessed the cost and performance of contingent and primary cfDNA screening. Results from most opted for contingent cfDNA screening because it can leverage the benefits of cfDNA screening at a substantially lower cost [16, 17, 28–30]. Our previous study found that even if the price of cfDNA screening fell to as low as $200 (CAD), contingent cfDNA screening would still more economical [17]. Both contingent and reflex cfDNA screening are two-step strategies. Contingent cfDNA screening has been used by a number of screening programs including in Ontario and British Columbia, Canada [18, 31]. With this strategy, women may drop out from follow-up cfDNA screening for various reasons and the strategy is unlikely to achieve its expected performance in the real-life clinical setting. Reflex cfDNA screening, on the other hand, has a higher chance to reach its performance potential because all eFTS positive women will receive a cfDNA test result. There is no need to recall patient for a second blood sample. Reflex cfDNA screening was first proposed by Wald et al. in 2013 [13]. A recent cohort study by the same group reported an observed DR of 95% and very low FPR at 0.02% [32]. Our results are in agreement with a recent cost and efficacy study comparing reflex and contingent cfDNA screening although we used eFTS as the first tier screening test and there are variations in the pathway of reflex cfDNA screening between the two studies [15].

Reported performance of cfDNA screening is usually based on tests with a result [33]. Sequencing based cfDNA screening has a no-call result rate of about 1.2% after repeat sampling [9]. After excluding women with no-call results, the actual DR is around 97% [33]. In primary cfDNA screening, if all women with a no-call result after repeat sampling have a diagnostic test, the total proportion of women requiring a diagnostic test is 1.3%, which is substantially higher than the contingent or reflex cfDNA screening models. Although cfDNA screening using RCA/Imaging solution technology has a lower reported no-call result rate [5], when used as a primary screening test, the numbers of women requiring genetic counselling and diagnostic testing is still much higher than with reflex cfDNA screening. No-call results and high costs are major obstacles to the implementation of a primary cfDNA screening strategy, especially for publicly funded screening programs which seek maximal benefits at the lowest cost.

In this study, we used eFTS as the first-tier test because it is the most commonly ordered aneuploidy screening test in Ontario currently. It was also identified in our previous study as the most cost-effective for contingent cfDNA screening [17]. A similar performance can be achieved with FTS by lowering the risk cut-off although the saving from removing PlGF and AFP will be offset by the increasing costs for genetic counselling and diagnostic testing [8]. Since with reflex cfDNA screening, all women receive screening results at the same time regardless of cfDNA screening being performed, the turnaround time of cfDNA screening becomes especially critical. Currently in Ontario, the turnaround time of sequencing- based cfDNA screening is about 10 days [22, 23]. New cfDNA screening technologies with a shorter turnaround time and lower no-call rate will make reflex cfDNA screening more feasible, especially if MMS and cfDNA screening can be performed in the same laboratory. In this study, we focused on trisomy 21 screening because the prevalence of trisomy 18 and 13 is significantly lower [19]. Most trisomy 18 and 13 affected pregnancies are also screen positive for trisomy 21. For example, when combining the screening algorithms for trisomy 21 and 18, 96% of trisomy 18 cases can be identified at a FPR of 5% for trisomy 21 and an additional FPR of 0.1% for trisomy 18 [34]. Assuming an additional eFTS positive rate of 0.2% for trisomy 18 and 13, with contingent or reflex cfDNA screening, the number of women requiring cfDNA screening or a diagnostic test due to a positive eFTS result for trisomy 18 or 13 would be small and the impact on cost marginal. The DR for trisomy 18 and 13 using the two strategies would be the same although a few more women would require a diagnostic test if sequencing-based cfDNA were used. The DR using primary cfDNA would be slightly higher if all women with a no-call result had a diagnostic test. However, more trisomy 18 or 13 affected pregnancies would be in the no-call result group especially with sequencing-based cfDNA [35]. In this study, we used the same unit cost for cfDNA screening regardless of technology, although RCA/Imaging solution technology is likely to be less costly [5]. By holding the cost of cfDNA tests consistent, we were able to assess the savings from using the reflex model and from the reduced no-call rate of RCA/Imaging solution technology. This also makes the results more relevant to other screening programs as the cost of cfDNA testing can vary between countries/screening programs. The savings from using a lower cost cfDNA test could be easily calculated by entering a new cfDNA unit cost in our model.

Lastly, both contingent and reflex cfDNA screening models permit the addition of pre-eclampsia screening. By adding a few maternal characteristics not currently collected for MMS, eFTS can identify 68% of the pregnancies at risk of developing early-onset pre-eclampsia at a false positive rate of 10% [36]. Since eFTS includes PlGF and PAPP-A, two biochemical markers used for pre-eclampsia screening, the cost of adding a screening algorithm for early-onset pre-eclampsia would be minimal. The screening performance for early-onset pre-eclampsia could be further improved by adding mean arterial blood pressure and uterine Doppler assessment and/or lowering the risk cut-off to identify more at-risk women for further testing [37].

Our study has some limitations. We assumed a 100% uptake rate of cfDNA screening, genetic counselling and diagnostic testing to model the best screening performance and to simplify comparisons. In reality, factors such as age, socio-economic status, ethnicity and religious beliefs can influence uptake rates [38, 39]. For example, the uptake of genetic counselling following a positive eFTS or high-risk cfDNA screening result may be very different. Women carrying pregnancies with ultrasound detected anomalies or at risk of another genetic condition may choose diagnostic testing over screening. In this study, we did not factor in the cost of temporary storage of samples for the possible cfDNA screening (about 3 days) with the reflex cfDNA screening approach. Since all MMS samples are stored until after delivery as per Ontario guidelines [40], the extra costs to the existing storage system should be minimal. For costs associated with each screening strategy, we did not include the cost for patients to participate in screening, e.g. time spent on blood draw or in genetic counselling. In addition, the costs of caring for infants with aneuploidy were not included in the study. Turnaround time of cfDNA screening in this study was based on local experience which can be variable across screening programs.

In this study, we compared screening strategies using sequencing based cfDNA screening and cfDNA screening with RCA/Imaging solution. While cfDNA screening with RCA/Imaging solution is promising for its simplified workflow, increased accessibility, high DR and low no-call result rate [5, 10, 24], the test is new and the number of published scientific studies on this technology is limited. Nevertheless, the results of this study can be generalized to other emerging cfDNA screening platforms with similar DR, FPR and no-call result rate that become available.

## Conclusions

In summary, our study compares performance, costs and timeliness of cfDNA screening results of contingent, reflex and primary cfDNA screening strategies for trisomy 21. Reflex cfDNA screening provides a similar DR for trisomy 21 to contingent cfDNA screening, with a final risk assessment available at an earlier gestation, and at a lower cost. It is more efficient to health-care system and pregnant women as only one patient visit is needed before a final screening result is generated. To introduce a reflex cfDNA screening strategy, adequate patient education is required. If sufficient counselling can be provided at the beginning of the aneuploidy screening process to support informed choice, including a discussion about the screening process, implications of test results and the available options, then reflex cfDNA screening is the optimal strategy for prenatal screening for common aneuploidies.

## Data Availability

The datasets used and/or analysed during the current study are available from the corresponding author on reasonable request.

## Abbreviations

AFP: α-fetoprotein
cfDNA: Cell-free fetal DNA
DR: Detection rate
eFTS: Enhanced first trimester screening
FPR: False positive rate
FTS: First trimester combined screening
GC: Genetic counselling
hCG: Human chorionic gonadotrophin
MMS: Multiple marker screening
NT: Nuchal translucency
PAPP-A: Pregnancy-associated plasma protein A
PlGF: Placental growth factor
RCA: Rolling Circle Amplification
